# Investigating GLP-1 sourcing preferences on social media and motivations for use: Survey with choice-based conjoint (CBC) exercise

**DOI:** 10.1371/journal.pone.0359937

**Published:** 2026-11-04

**Authors:** Michael Robert Haupt, Staci Meredith Weiss, Alec J. Calac, Sydney Salazar, Meng Zhen Larsen, Tim K. Mackey

**Affiliations:** 1 Global Health Program, Department of Anthropology, University of California, San Diego, California, United States of America; 2 Global Health Policy & Data Institute, San Diego, California, United States of America; 3 University of Roehampton, School of Psychology, London, United Kingdom; 4 School of Medicine, University of California, San Diego, California, United States of America; 5 S-3 Research LLC, San Diego, California, United States of America; PLoS ONE, UNITED STATES OF AMERICA

## Abstract

The growing use of glucagon-like peptide 1 (GLP-1) drugs such as *Ozempic* for weight loss interventions has led to increased concerns about inappropriate use, supply shortages, and high-risk sourcing. However, little is known about the influence of online marketing strategies on purchasing preferences for GLP-1 products. This study uses a survey with a Choice-Based Conjoint (CBC) exercise to assess features of social media posts that are most influential for intent to purchase GLP-1 products and identify motivations for use. Participants were recruited based on current use of GLP-1 drugs, previous use, or considering use (n = 242). Utility scores from the conjoint analysis quantified the influence of tested features. Conjoint findings showed greater preference for posts that feature branded semaglutide (e.g., Wegovy)(15.38, SD = 45.29), display products in packaging (47.49, SD = 46.72), have accredited digital pharmacies as the seller (25.16, SD = 47.31) (e.g., Amazon Pharmacy), include medical endorsements for diabetes treatment (13.16, SD = 49.04), and offer a free consultation (13.18, SD = 32.00). Analysis was conducted on survey questions measuring GLP-1 purchasing channels and motivations for use. Over half of participants (51.7%) reported that social media content influenced their perceptions of GLP-1 drugs and 45.8% (n = 65) of GLP-1 users reported purchasing product from high-risk sources (i.e., personal network or individual sellers online). Exploratory analysis shows a possible association between self-reported high-risk sourcing and reliance on GLP-1s for medical conditions. Overall, our findings identify influential attributes of GLP-1 social media ads (i.e., branding and visual display of products) and suggests greater support is needed for online moderation efforts to prevent high-risk purchasing activity and increase safe access to GLP-1s for those with medical needs.

## Introduction

The growing popularity of glucagon-like peptide 1 (GLP-1) drugs such as *Ozempic* (semaglutide), *Wegovy,* and *Mounjaro* (tirzepatide) for weight loss interventions has led to increased concerns surrounding inappropriate prescribing, off-label use, and supply shortages for those who rely on them medically [1] (e.g., for diabetes treatment [2]). Direct-to-consumer advertising (DTCA) of GLP-1s is widespread, including promotion on social media platforms and by influencers, telemedicine/telepharmacy ads for compounded versions, manufacturer websites promoting branded GLP-1s, and even access via illegal online pharmacies [3–5]. Similar to other drug types [6–13], there is appetite for digital sources of reliable GLP-1 products with less stringent treatment eligibility criteria and ease of access [14].

Access to GLP-1s varies and can be impacted by factors such as insurance coverage, resource deprivation indices, and racial identity [15,16]. Other relevant factors include access to pharmaceutical agents outside of the traditionally-regulated prescription pipeline (e.g., online purchasing), which may increase the likelihood of patient adverse events and raise concerns about exposure to products of lower quality or that are falsified [4,17]. The ubiquity and convenience of online platforms make them appealing options for sourcing prescription products [6]. For GLP-1 products, where there is high demand that often exceeds supplies [5,18], people may search online for unregulated sellers if they experience barriers sourcing from legitimate channels. A surveillance study of social media discussion about GLP-1s shows how uneven access relates to patient population and demand characteristics. Specifically, this study found that demand for GLP-1s in online forums focused on off-label use (e.g., PCOS, eating disorders, influencers) and identified patient concerns regarding insurance coverage, adverse effects, medication administration, and informed consent discussions [19], which may be more appropriate for discussion with a physician than a digital consultation.

The popularity of GLP-1 discussions in online forums indicates a need to examine the most commonly used sources for obtaining information on GLP-1s. There is equally a need to conduct studies that specifically examine consumer GLP-1 sourcing behavior and how digital media can mediate access to this widely used class of medications. As promotion and channels of access continue to evolve for GLP-1 drugs, the objective of this study was to conduct an online survey and Choice-Based Conjoint (CBC) exercise to examine online sourcing preferences for GLP-1s and identify underlying motivations and influences for use.

## Materials and methods

### Overview of conjoint analysis

We used conjoint analysis to assess which features of social media posts advertising GLP-1 drugs are most influential on purchase intent. Conjoint analysis is an experimental approach developed in the field of mathematical psychology and is often employed by market researchers to quantify preferences for products or services among consumers (see [20,21] for a more comprehensive review). A conjoint study mimics complex decision-making processes that require people to “consider jointly” multiple attributes by choosing hypothetical product alternatives. When designing a conjoint exercise, a product is thought of as being comprised of attributes (e.g., Color) that has several possible levels (e.g., Blue, Red). Respondent preferences are revealed as part-worth utility scores based on the levels chosen most often in the conjoint exercise.

The use of conjoint analysis for health-related research has grown in recent years [22]. Pharmacology researchers and medical scientists have used conjoint analysis to evaluate patient preferences for HIV prevention medication [23], vaccine treatments [24], and disease modifying therapies [25]. Within the context of online marketing, recent work employs conjoint analysis to assess how online users evaluate safety of social media posts that advertised illegal access to controlled substances [7]. An important advantage of conjoint analysis is that it can reduce social desirability bias in survey responses and identify covert attitudes [26–28]. As the present study requires respondents to disclose sensitive information related to drug purchasing preferences, conjoint analysis was chosen to quantify the appeal of GLP-1 marketing features while mitigating concerns from social desirability biases. While previously used to examine health-related decision-making processes, there is currently no work that uses conjoint analysis to assess purchasing preferences for GLP-1s within the context of social media.

### Conjoint exercise levels and attributes

Conjoint analysis was used to assess influential features of social media posts on intention to purchase GLP-1 drugs. TikTok was tested in the current study due to its specialization in visual marketing (such as targeted ads), popularity among younger users, and recent work showing GLP-1 advertising and discussion on the platform [29–31]. The following attributes were tested based on influential features of social media posts identified in previous work [7,32,33]: *Brand Type*, *Seller*, *Visual Display*, *Product Positioning*, and *Caption Text*. See Table 1 for full description of attributes/levels and rationale for testing in the present study. As we are interested in assessing influential effects from marketing factors and not economic restraints, we did not include price as an attribute.

**Table 1:** Attributes and levels tested in conjoint exercise.

Attribute	Levels	Description
**Brand Type**	1. Branded Semaglutide – Ozempic 2. Other Branded Semaglutide (ex- Wegovy) 3. Pharmacy Branded Semaglutide (ex- Hers/Hims Compounded Semaglutide) 4. Branded Tirzepatide (ex- Mounjaro)	Assesses the extent that GLP-1 branding influences intent to purchase. As Ozempic is currently the leading brand in the GLP-1 product category [34,35], this brand was tested separately to quantify and compare how much participants value the Ozempic brand relative to other Semaglutide products. Tirzepatide products (e.g., Mounjaro) were also tested as they have experienced a similar demand trajectory to Semaglutide for off-label use as weight loss treatments.
**Seller**	1. Manufacturer (LilyDirect) 2. Digital pharmacy (Amazon Pharmacy, HealthWarehoise) 3. Individual white female in medical clothing	Tests influence of post author. Specifically, we assessed the extent that participants value that the seller is from an organization or individual user. Levels include: the manufacturer (LillyDirect), digital pharmacies (Amazon Pharmacy and HealthWarehouse), and an individual white female dressed in medical clothing (peer-like source in professional attire).
**Visual Display**	1. Product packaging 2. Post author	Test influence of main image used in the mock posts, as previous work indicates that images used to display drug products in social media posts are influential credibility markers [7]. The two levels test whether the image shows GLP-1 products in packaging or displays the post author. The Product packaging level is dependent on the level assigned by the *Brand Type* attribute (e.g., Ozempic packaging when *Brand Type* = Ozempic). For the Post author level, logos for organizations were displayed if *Seller* = Manufacturer and website listings for products were used if *Seller* = Online Pharmacy.
**Product Positioning**	1. Medical endorsement (diabetes) 2. Medical endorsement (weight loss) 3. Celebrity endorsement (weight loss) 4. No text graphic (control)	Tests variations of text displayed in a graphic that is overlayed on the main image. The tested levels were selected based on the fact that GLP-1 products are used for both diabetes and weight loss treatments, and have been promoted by medical practitioners and celebrities for both U.S. Food and Drug Administration (FDA) approved indications and off-label use [30,31,36,37]. Levels include: medical endorsements that mention diabetes treatment (e.g., “Clinically tested for treating Type-2 diabetes”), medical endorsements that mention weight loss (e.g., “Doctors say this is a game changer for your weight loss journey”), celebrity endorsements that mention weight loss (e.g., “This is a must have for Kim Kardasian’s weight loss routine!”), and no text graphic as a control comparison.
**Caption Text**	1. Sale promotion only 2. Sale promotion + free consultation 3. Sale promotion + no prescription required	Tests variations of text included in the caption of the post on purchase intent. Levels test whether posts include a sale promotion only (e.g., “Message us to purchase Ozempic”), a sale promotion with an offer to schedule a free consultation, or a sale promotion that mentions no prescription is required for purchase. These levels were selected to assess how factors associated with high risk purchasing (e.g., no prescription required) influences consumer decision-making.

This study is divided into 2 parts. In part 1, participants completed the conjoint exercise. In part 2 respondents answered questions about GLP-1 purchasing habits and motivations for use.

### Data collection

Participants (n = 242) were recruited online during November 2024 on Amazon Mechanical Turk (MTurk). Sample size was determined to have sufficient statistical power to observe main effects from tested conjoint design. MTurk is traditionally used for recruiting human respondents to improve training datasets for artificial intelligence software [38] and is widely used as a sampling source in social science research [38,39] and more recently for surveying online drug purchasing preferences [7,40]. Participants were eligible based on the condition of previously purchasing health products online, and current, previous, or ‘considering’ use of GLP-1 drugs. Respondents were removed if they failed at least 2 data quality checks (e.g., failing attention checks).

Ethics approval for this study was granted by the University of California, San Diego (IRB protocol number: 810321) and all methods and protocols were carried out in accordance with IRB guidelines. MTurk workers completed the survey anonymously and were compensated based on standard survey-taking rates on the platform. Our data collection approach complied with the terms and conditions for using the MTurk platform and informed consent was obtained from all participants. Specifically, participants were shown the consent form and asked whether they agree to participate before starting the survey. See **S1 Methods** for additional details on recruiting criteria and the following Open Science Framework (OSF) depository for a report on the tested conjoint design and the data used in analysis: https://osf.io/56cv4/?view_only=982be7612cff49058aa93198fcbe31ec. Please reach out to the first author for inquiries about the dataset used in this study.

### Conjoint design and statistical analysis

The variant of conjoint analysis used in the current study is called Choice-Based Conjoint (CBC). In total, 288 mock posts were created based on every possible combination of tested attributes (*Brand Type* (4 levels) x *Seller* (3 levels) x *Visual Display* (2 levels) x *Product Positioning* (4 levels) x *Caption Text* (3 levels) = 288). In order to create realistic stimuli, posts were produced using an online mock social media post generator (zeoob.com). Each participant evaluated 36 posts selectively chosen to assure sufficient exposure for each attribute. Participants were shown 2 posts at a time for 18 rounds and asked to select the one they would be most likely to purchase from. Participants were also given the option “None: I wouldn’t choose either of these” in each round. See Fig 1 for examples of the mock social media post and conjoint purchasing task.

**Fig 1:**
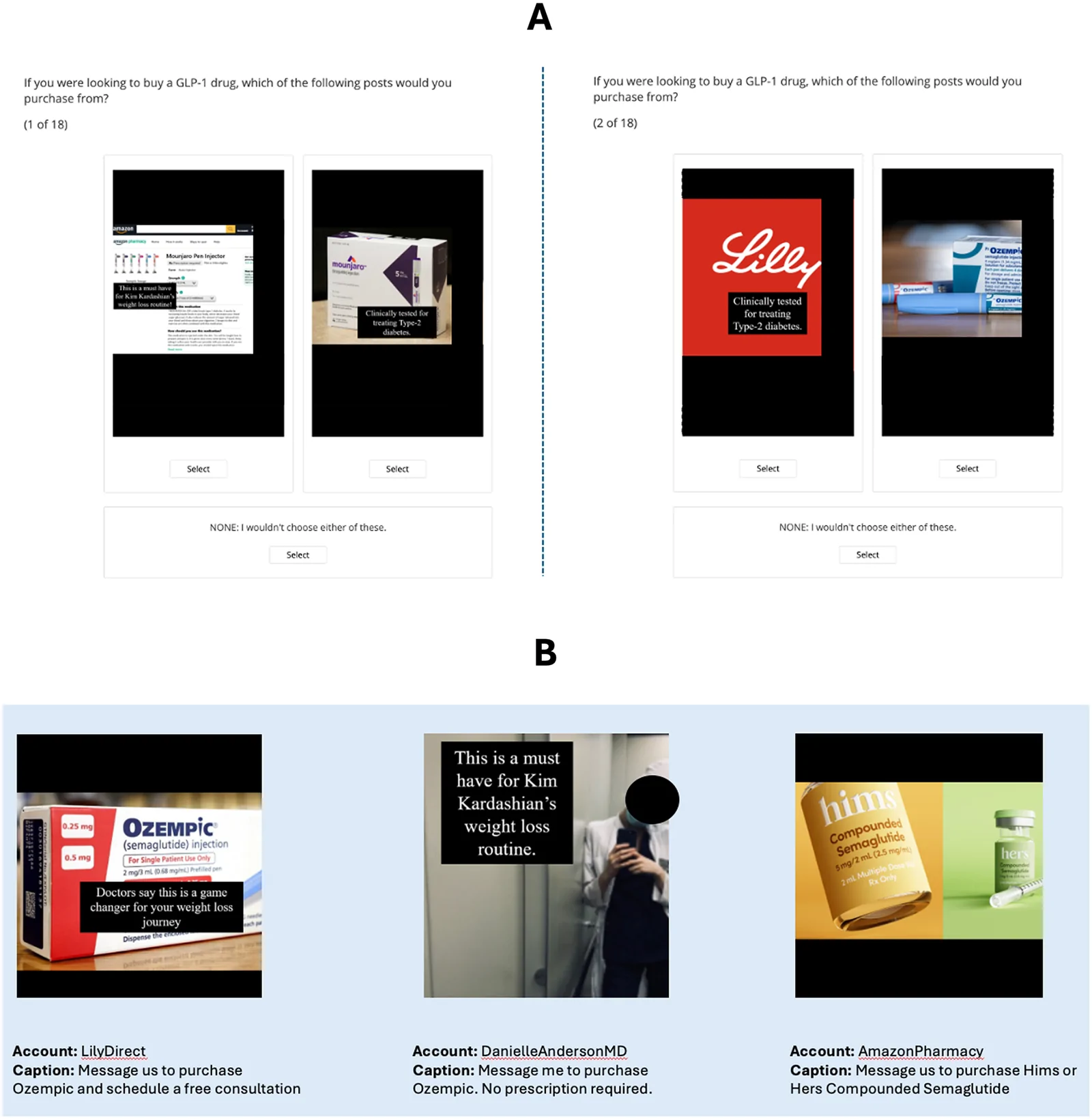
Examples of Conjoint Exercise (A) and Mock Social Media Posts (B). **Levels displayed in bottom of figure (B)**: (*Left*) Branded Semaglutide – Ozempic, Manufacturer (LillyDirect), Drug in packaging displayed, Medical Endorsement – Weight Loss, Sale Promotion + Offers Consultation (*Center*) Branded Semaglutide – Ozempic, White female in medical clothing, Seller displayed, Celebrity Endorsement – Weight Loss, Sale Promotion + No prescription required (*Right*) Pharmacy Brand Semaglutide, Digital Pharmacy, Drug in packaging displayed, No Graphic Text, Sale Promotion Only. NOTE: The posts in this figure were heavily edited to avoid copyright violations from showing TikTok’s user interface. These changes are for publication only and participants did not see these edits in the conjoint exercise.

Each tested level in the conjoint exercise is used as an independent variable for multinomial logistic regression modeling. The program Lighthouse Studios developed by Sawtooth Software produced part-worth utility scores (i.e., beta coefficients) using hierarchical bayes estimations that account for choices from individual respondents and the sample average. Utilities per attribute are averaged and scaled to be normalized “zero-centered diffs,” which cause the utilities to sum to 0 within each attribute. When interpreting results from conjoint analysis, the levels with the highest average utility scores indicate higher preference. Importantly, only utility scores of levels within the same attribute can be compared, but not across attributes. While levels cannot be directly compared across attributes, it is possible to assess which attributes overall are weighted most heavily in respondent decision-making processes by using importance scores, which are computed for each attribute by taking the difference of the range in utility values and then dividing it by the sum of the differences in ranges across all attributes.

In our exploratory analysis, we report survey results from questions about GLP-1 attitudes, purchasing habits, and motivations for use. Our questions were adapted from previous research examining online drug sourcing [7,40]. We also examine differences between those who report purchasing a GLP-1 product from a high-risk channel compared to those who have only purchased from legitimate channels. We define high-risk channels as sellers who are generally not authorized to sell GLP-1 products, which have a higher likelihood of selling substandard, falsified, or counterfeit products due to lack of regulatory oversight. Specifically, this included sellers from one’s personal network, or individual sellers from an online marketplace, social media platform, or messaging application. Channels that require a prescription from a licensed healthcare provider and operate under regulatory supervision such as licensed pharmacies, accredited digital pharmacies, or telehealth providers were classified as legitimate, though there are limitations to this classification approach as mentioned in the discussion.

## Results

Our sample of MTurk respondents consisted of 58.7% (n = 142) current GLP-1 users, 11.6% (n = 28) prior users, and 29.8% (n = 72) considering to use GLP-1s. The sample skewed male (72.7%) with a mean age of 34.71 (SD = 9.61, min = 24, max = 80). Up to 74.38% report an annual household income of at least $50,000 and 75.6% have a bachelor’s degree. Further, 86.4% identified as White, 2.1% as Black, 9.5% as Asian, 3.3% as American Indian, and 35.5% as Hispanic. See S1 Table, S2 Table, S3 Table, and S4 Table for full demographic details of sample.

Fig 2, Fig 3 and Table 2 show the results from the GLP-1 conjoint exercise. Among the tested attributes, *Brand Type* (23.11, SD = 9.12) and *Visual Display* (22.68, SD = 13.97) were ranked the most influential for purchasing intent while *Caption Text* was the lowest (13.29, SD = 7.40). Within *Brand Type*, the levels for *Other Branded Semaglutide* (e.g., Wegovy) (15.38, SD = 45.29) was ranked the highest in preference followed by *Branded* *Semaglutide – Ozempic* (8.93, SD = 41.54). *Pharmacy Branded Semaglutide* (e.g., His/Hers) was ranked the lowest (−15.38, SD = 52.99). Within the *Seller* attribute, *Digital Pharmacy* was ranked the highest (25.16, SD = 47.31) while *Manufacturer* was lowest (−14.81, SD = 41.82). For *Visual Display*, *Drug in Packaging* (47.49, SD = 46.72) was ranked higher than *Picture of Seller*. For *Product Positioning*, *Medical Endorsements for Diabetes* treatment (13.16, SD = 49.04) was ranked highest while *Celebrity Endorsement for Weight Loss* was ranked lowest (−15.90, SD = 50.37). Lastly, *Caption Text* that offered a consultation was ranked the highest (13.18, SD = 32.00) compared to *Sales Promotion Only* (−0.61, SD = 30.45) and mentioning no prescription is required (−12.56, SD = 29.25).

**Table 2:** GLP-1: Average importance scores and utility scores (zero-centered diffs) for tested attributes and levels.

*Attribute*	*Average Importances* *(Std Dev)*	*Levels*	*Average Utilities* *(Std Dev)*
***Brand*** ***Type***	23.11 (9.12)	Branded Semaglutide – Ozempic	8.93 (41.54)
		**Other Branded Semaglutide (ex- Wegovy)**	**15.38** **(45.29)**
		Pharmacy Branded Semaglutide (ex- Hers/Hims Compounded Semaglutide)	−15.78 (52.99)
		Branded Tirzepatide (ex- Mounjaro)	−8.53 (44.41)
***Seller***	19.26 (9.16)	Manufacturer (LillyDirect)	−14.81 (41.82)
		**Online Pharmacy (Amazon Pharmacy, HealthWarehouse)**	**25.16** **(47.31)**
		White female in medical clothing	−10.35 (37.61)
***Visual Display***	22.68 (13.97)	**Product packaging (see Brand Status)**	**47.49** **(46.72)**
		Post Author	−47.49 (46.72)
***Product Positioning***	21.67 (11.63)	**Medical Endorsement – Diabetes**	**13.16** **(49.04)**
		Medical Endorsement – Weight Loss	0.50 (39.42)
		Celebrity Endorsement – Weight Loss	−15.90 (50.37)
		No Graphic Text	2.24 (45.96)
***Caption*** ***Text***	13.29 (7.40)	Sale promotion only	−0.61 (30.45)
		**Sale promotion + Offers consultation**	**13.18** **(32.00)**
		Sale promotion + No prescription required	−12.56 (29.25)

***Note***: **Bold and shaded cells** indicates level with the highest utility score within the attribute. Model Statistics: Number of iterations before using results = 10,000; Number of draws per respondents = 10,000

**Fig 2:**
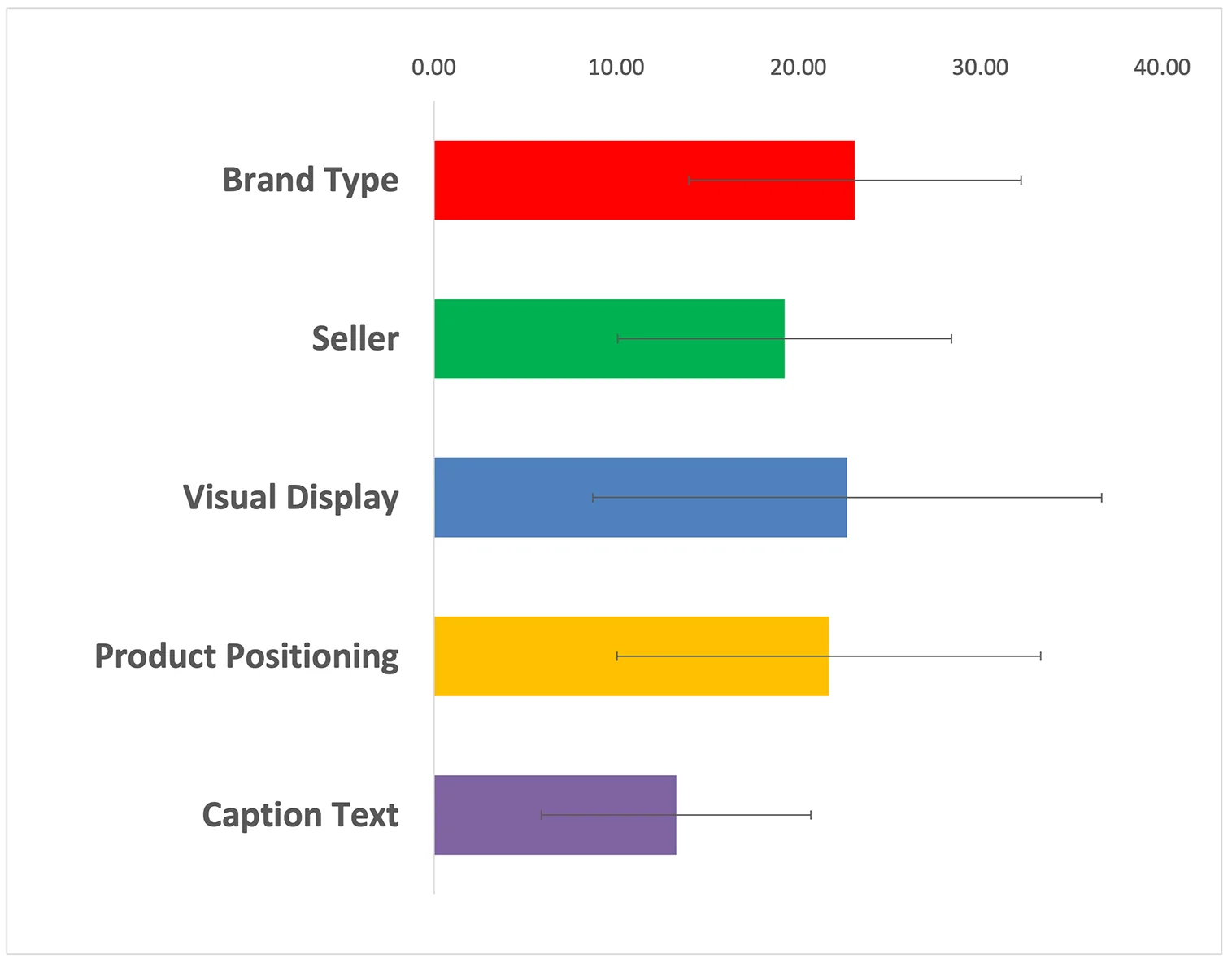
Average importance scores for tested attributes (error bars = +/- 1 SD).

**Fig 3:**
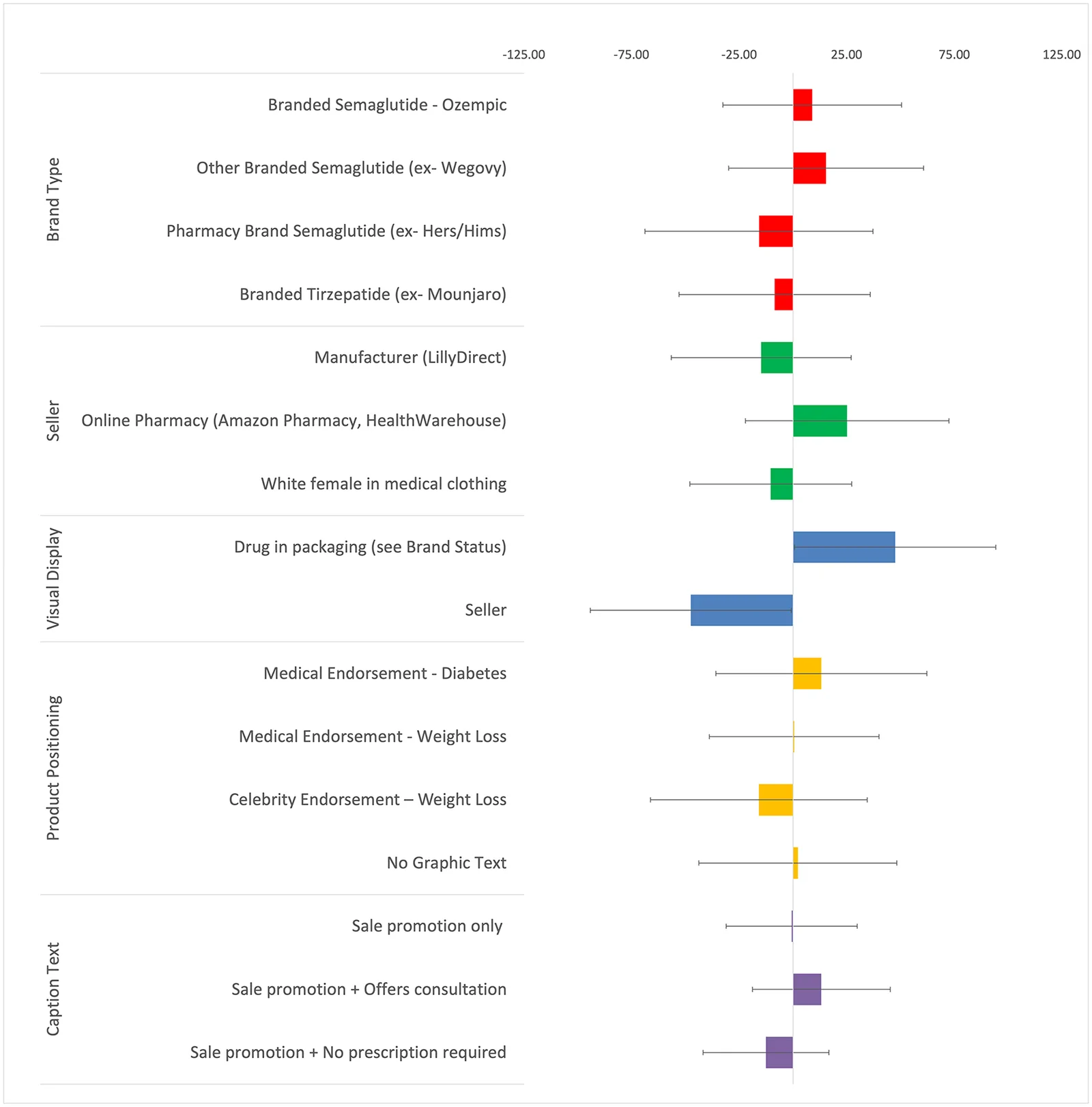
Average utility scores for tested levels (error bars = +/- 1 SD).

Out of all the conjoint rounds that respondents were shown, 5.5% selected “None” (Average Utility score = −468.53). This indicates that respondents preferred the tested level combinations over not making a GLP-1 purchase. We further examined if age and gender showed significant differences in level preference. Spearman correlations were conducted to observe differences for age and Mann-Whitney U Tests for gender due to the non-parametric nature of the utility scores. Benjamini-Hochberg method was used to correct for multiple comparisons. There were no significant effects observed for age and gender on preferences for individual levels. The lack of significant effects suggest that age and gender do not produce major differences in susceptibility to online GLP-1 marketing for the tested levels.

### Purchasing Preferences

Results from the survey following the conjoint exercise found that almost 2 in 3 (64.1%, n = 170) current and past GLP-1 users preferred branded GLP-1 injections (e.g., Ozempic) while 25.3% preferred branded GLP-1 pills (e.g., Rybelsus). Only 10% of GLP-1 users preferred generic brand (e.g., compounded versions) while only 0.6% expressed no preference for branded products. Exploratory analysis suggests that those considering use of GLP-1s may have greater preference for generic GLP-1s compared to users (36.1% Consider vs 11.8% Users, p < .001, OR=0.236, X^2^(1)=19.47) and may be more likely to have no GLP-1 brand preference (8.3% Consider vs 0.6% Users, p < .001, OR=0.065, X^2^(1)=10.8) (S5 Table).

Current GLP-1 users report purchasing from at least 2 channels on average (SD = 1.06, max = 5) and 37.3% (n = 53) users sourced exclusively from 1 channel. Most current users (83.1%, n = 118) purchased from at least 1 online channel. As shown in Fig 4, the generic category of an “online pharmacy” (37.3%) was the most frequently reported purchasing channel among GLP-1 current users followed by individual sellers from online marketplaces (33.8%), and websites of pharmacy/drug stores (33.1%). Around half of current users (45.8%, n = 65) reported purchasing product from individual sellers online or personal networks, which have higher risk of selling substandard or falsified products compared to legitimate channels (e.g., pharmacy/drug store, compounding pharmacies, telehealth providers, etc). Exploratory analysis compares attitude differences between high-risk and low-risk purchasers. The non-parametric Mann-Whitney U Test was used due to the non-normal distribution of the attitude scales. Findings suggest that those who purchased from high-risk channels also had a higher tendency to report relying on GLP-1 drugs to help treat a medical condition compared to those who did not purchase from those sources (mean = 4.09 vs 3.69, p < .05, Mann-Whitney U Test = 1976, Z = −2.27). However, this effect no longer remains significant after controlling for multiple comparisons (Benjamini-Hochberg).

**Fig 4:**
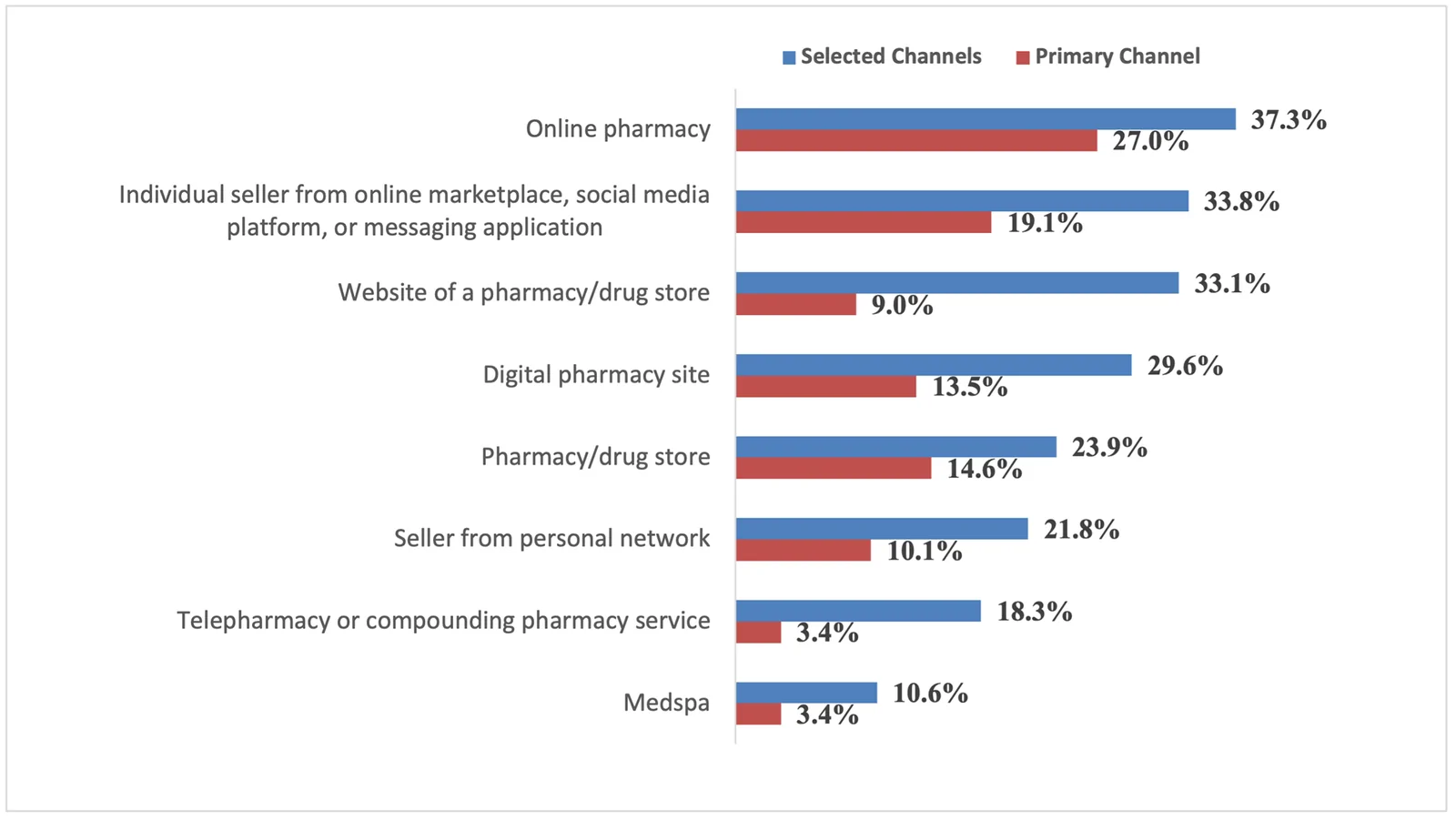
Channels used to Purchase GLP-1 Drugs among Current Users (n = 142). **Question Text:** Where do you typically purchase GLP-1 drugs? Please select all that apply*.; [If multiple channels selected, n = 89]* Of those types of options that you just selected, where do you primarily purchase GLP-1 drugs?

As shown in S1 Fig, specific websites/platforms used by online purchasers include sites for legitimate and accredited digital pharmacy vendors and national pharmacy retailers such as Amazon Pharmacy (64.4%, n = 76), Walmart Pharmacy (46.6%, n = 55), CVS (42.4%, n = 50), and HealthWarehouse (31.4%, n = 37). However, results also show that messaging apps such as WhatsApp (62.7%, n = 74) and networking sites such as Twitter (currently “X”) (52.5%, n = 62) and YouTube (50.8%, n = 60) were reported by a majority of online purchasers for sourcing GLP-1 products. Other higher-risk platforms for GLP-1 purchasing were reported at a lower frequency, which included Google Groups (20.3%, n = 24), Signal (11.0%, n = 13), Discord (9.3%, n = 11), and Craigslist (5.9%, n = 7). It is worth noting that these findings are unable to reveal how often these platforms or messaging applications were used to purchase GLP-1s or the nature of these sales transactions, as purchasing from social media platforms could include transactions that are both legal (e.g., ad from a legitimate vendor [41]) or illicit (e.g., purchasing GLP-1 product from another user in the comment section or from an unregulated or unauthorized online pharmacy [8,9,42]).

### Motivations for Using GLP-1s

Participants reported 1.9 motivations on average (SD = 1.2) for wanting to use GLP-1 products. As shown in Fig 5, participants were motivated to use GLP-1 products to control weight after: long-term struggles with weight (30.3%, n = 73), recent weight gain unrelated to medical condition (27.8%, n = 67), and weight gain from a recent medical condition (e.g., pregnancy, SSRI use) (24.1%, n = 58). Almost half of participants (48.8%, n = 119) only mention weight-related reasons for taking GLP-1s while 14.8% (n = 36) report only using GLP-1s to control blood sugar. Over a third of participants (36.5%, n = 89) reported both weight and blood-related reasons.

**Fig 5:**
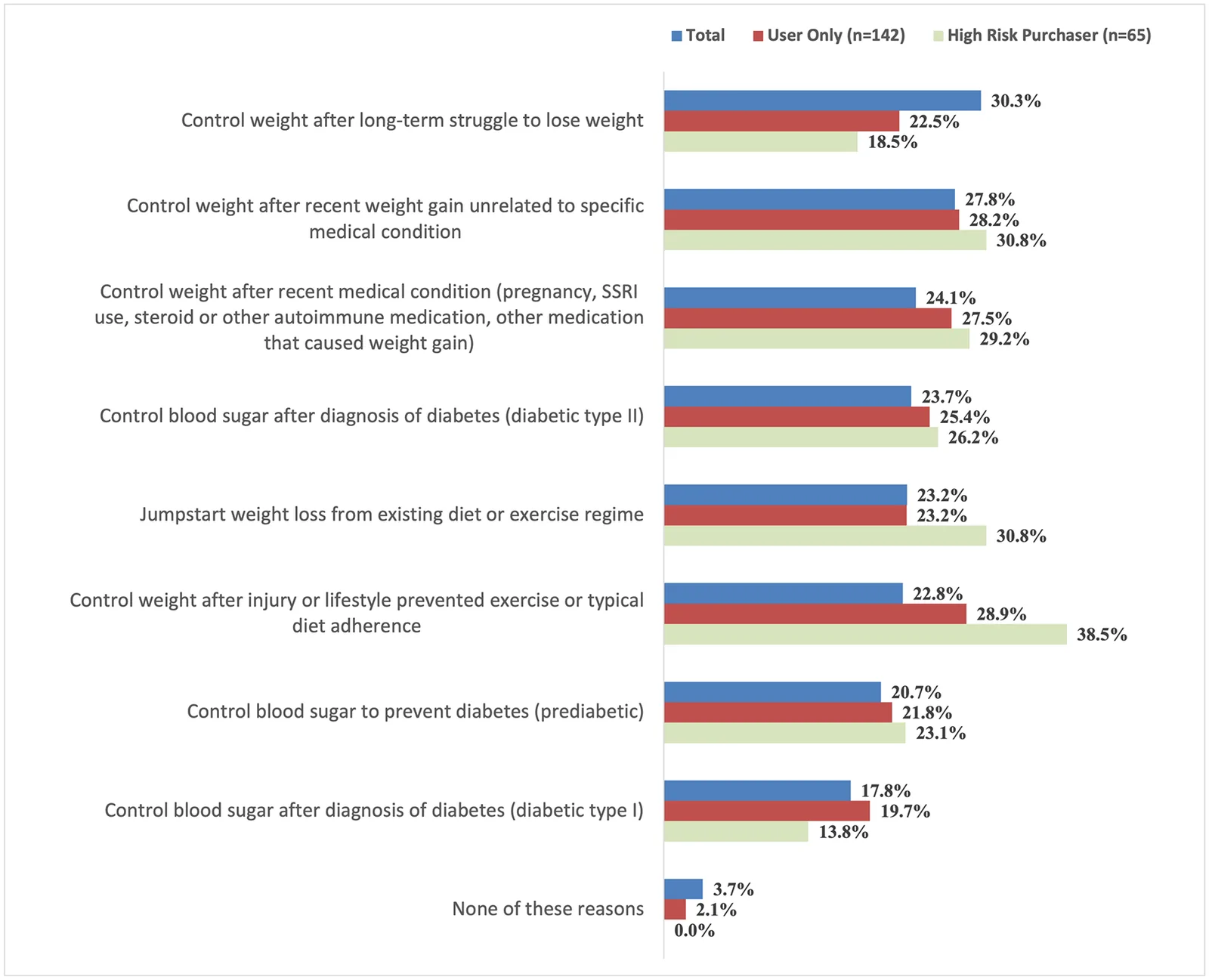
Motivations for Using GLP-1 Products. Question Text: Are you interested in using GLP-1 drugs in order to (Check all that apply) ***Note*:** Base for total is n = 241 due to missing data from 1 respondent.

Chi-square tests and Z-tests for pairwise comparison from our exploratory analysis suggest GLP-1 users were more likely to report motivations to ‘control weight after an injury or lifestyle prevented exercise’ compared to those only considering use (28.9% vs 9.9%, p < .01, OR=3.60, X^2^(1)=9.60). Conversely, findings suggest those considering use may be more interested in GLP-1s to address long-term difficulty losing weight compared to current users (40.8% vs 22.5%, p < .01, OR=.51, X^2^(1)=5.31). Chi-square effects remained significant (p < .05) when adjusting for multiple comparisons (Benjamini-Hochberg). Users who purchased from high-risk channels may be more interested in GLP-1s to ‘control weight after injury or lifestyle preventing exercise’ compared to users who did not purchase from those channels (38.5% vs 20.8%, p < .05), however, this effect did not remain significant after applying the Benjamini-Hochberg correction.

### GLP-1 Influences

Medical professionals in-person (52.9%) and on social media (51.7%) were selected most often as influencers of GLP-1 perceptions, followed by discussions with friends and family members (49.6%), and magazines (43.4%) (**S2 Fig**). Media sources were also influential: almost 1 in 3 were influenced by ads on TV or online (34.7%), posts on social media sites such as Facebook (33.5%), TV talk shows such as Dr. Phil (32.2%), and online influencers who are not medical professionals (31.4%). *Instagram* (78.7%) was the most influential platform for GLP-1 perceptions followed by *Facebook* (58%), *YouTube* (42%), and *TikTok* (34.8%) among participants influenced by at least 1 online source (n = 207)(**S3 Fig**).

## Discussion

The findings from our conjoint experiment of participants who currently use, previously used, or ‘considering’ use of GLP-1s show that branding and images are the most influential features on purchase intent when advertising GLP-1 products in social media posts compared to descriptive features such as caption text. Despite increased efforts from online marketers and sellers to capitalize on supply shortages of branded GLP-1 products by promoting compounded alternatives [5,43], our conjoint results show that users and potential users still show greater preference for recognizable semaglutide brands (e.g., Wegovy). Our results are consistent with findings from a previous conjoint study, which found that drug product packaging was the most important attribute for conveying safety from social media posts selling illicit substances [7]. This provides converging evidence suggesting that visual display and branding may be credibility signals for advertising drug products on social media across substance classes, although further work is needed to verify this generalization. Other characteristics of posts that had a higher likelihood of purchase include: the seller being an accredited digital pharmacy (e.g., Amazon Pharmacy), the product being displayed in drug packaging, the caption including a medical endorsement about effects on diabetes, and the caption text offering a free medical consultation. The implications of these findings are promising, as features that signal a high-risk purchase (e.g., caption text mentioning “no prescription is required”) were preferred less by participants. Likewise, levels that signaled approval by health authorities such as being sold by a legitimate pharmacy (compared to individual users) and graphics with medical endorsements (compared to celebrity endorsements) had a greater influence on purchase intent.

While the conjoint findings suggest participants have favorable attitudes towards medical institutions, it is worth cautioning that medical authority signals (e.g., phrases claiming to be “endorsed by doctors”) can be perversely used on social media to promote and lend credibility to health products not supported by scientific testing [44]. In our conjoint experiment, we found that caption text mentioning a “free consultation” was appealing to participants, which has the potential to be abused by unscrupulous sellers. A consultation given by a healthcare provider would in fact benefit users by allowing them to discuss their concerns with a professional for using GLP-1s and ensuring medically appropriate use. However, high-risk sellers could offer “free consultations” to signal medical legitimacy and personalized care, which can alleviate anxiety associated with purchasing from a high-risk channel and reduce friction from the purchasing processing by eliminating costs and effort needed to obtain a prescription from an authorized healthcare provider. Even consultations that only consist of online questionnaires or automated responses can create an illusion of professional oversight and safety. The use of the word “free” can also tap into well-documented psychological biases such as the “zero-price effect” where consumers disproportionally value free offerings [45,46], which would further promote the appeal of high-risk purchasing. Overall, the strategies used to convey credibility to products need to be accounted for in order to design effective interventions against high-risk sellers.

Despite greater preferences for features that signal medical authority, survey results following the conjoint exercise show almost half of current users (45.8%) report having purchased from a high-risk channel (i.e., individual sellers online or personal network). Online platforms such as social networking sites (e.g., YouTube, X) and messaging apps (e.g., WhatsApp) are also popular sourcing options. Exploratory analysis further shows that users who purchased from high-risk channels may be more likely to need GLP-1s to address medical conditions compared to users who only purchased from legitimate channels. This suggest participants with medical reliance on GLP-1 drugs might be pressured to purchase from personal networks and high-risk vendors on social networking sites despite preference for branded products from authorized sources (e.g., accredited digital pharmacies). In an era of targeted pharmaceutical and telepharmacy advertising via social media by both authorized and high-risk vendors [41,47–49], this could make users seeking legitimate products vulnerable to predatory sellers, who might not adhere to the same standards of drug safety, compound product testing, patient health monitoring and eligibility criteria screening as regulated sources [4,17].

In fact, recent work shows evidence of an emerging unregulated supply chain for GLP-1s with many offers leading to substandard and falsified products marketed and sold direct-to-consumer without a prescription [4]. Further, both the U.S. Food and Drug Administration and World Health Organization warn consumers about the presence of counterfeit GLP-1s in the U.S. drug supply chain and in other countries [50]. In the present study, the generic category of online pharmacies were the most frequently reported source for GLP-1s, which is concerning as some in this category may operate unregulated and sell falsified products (according to the National Association of Boards of Pharmacy, nearly 95% of websites offering prescription-only drugs online operate illegally [51]). These patient risks are likely to be accentuated as certain semaglutide drug shortages have been resolved according to the FDA as of February 2025 and compounded versions are now being phased out [52]. This is likely to cause consumer/patient confusion as previous compounded versions become unavailable, potentially leading to a rise in demand from high-risk channels. GLP-1 users may also be drawn to high-risk sellers due to cheaper prices, as 54% of adults who have taken GLP-1 drugs say it was difficult to afford the cost, including 53% of insured adults [53].

When considering motivations for using GLP-1s, our findings are consistent with recent work. A national survey shows that 62% of adults report taking GLP-1s to treat chronic conditions such as diabetes and 38% state losing weight as their main motivation for use [53]. Our study shows similar proportions, although our sample skews more towards reporting weight-related reasons (48.8% vs 38%). This difference may be attributed to our screening criteria, as we required participants to have ever purchased a health product online. GLP-1 users driven by weight-related reasons may be younger and more likely to use the internet to source health products, while those who never made an online health purchase may be more risk-averse, older, and more likely to have a chronic condition.

We also identified traditional media and social media as influential sources in shaping perceptions of GLP-1 products. Since social media is widely used to share personal experiences with GLP-1s in conjunction with traditional and expanding forms of DTCA (e.g., branded patient portals, DTCA from telehealth providers, etc.) [54], researchers should continue to monitor consumer sourcing behavior and assess its potential impact on health behavior and outcomes. Our results in **S6 Table** and **S1 Text** further found that GLP-1 users were significantly less likely to report being knowledgeable about GLP-1 drugs compared to those considering use, implying an educational need for current GLP-1 users on their prescription.

### Policy Implications

The majority of GLP-1 users in our study (83.1%, n = 118) purchased from at least 1 online channel, with over a third reporting to have purchased from an unregulated online seller. Popular online platforms include legitimate digital pharmacies (e.g., Amazon Pharmacy, CVS) and more troubling channels such as messaging apps (e.g., WhatsApp) and networking sites (YouTube, X) where there is a higher chance to purchase unregulated product from a high-risk seller. Social media is also a popular source of information about GLP-1s: over half of participants mention that medical professionals on social media influenced their perceptions of GLP-1 drugs and almost a third were influenced by non-medical professionals. The frequent use of online platforms to purchase and learn about GLP-1 products indicate the need for policies that promote accurate information about the benefits and risks of using GLP-1s and limit incentive to purchase unregulated product.

Increasing supply chain and accessibility to GLP-1 products can mitigate use of unregulated products, as limiting authorized access could lead to illicit sourcing. Enhanced moderation policies on social media platforms such as X, YouTube, and TikTok can also prevent high-risk purchases by flagging or removing posts with characteristics indicating the seller is not a legitimate vendor. This could include posts advertising GLP-1 products that use the phrase “no prescription required” or posts that advertise GLP-1 products but are from individual accounts not linked to an authorized seller. As mentioned previously, offerings such as “free consultations” can lend medical credibility to high-risk vendors and reduce anxieties from making a purchase. To counter this, regulators could require that anyone offering consultations meet specific standards based on qualifications and thoroughness of the consultation process (e.g., ensuring an appropriate physician-patient relationship and interaction), which would need to be explicitly stated in marketing materials.

Educational campaigns can further ensure that accurate information related to GLP-1 products is accessible across online spaces. Based on the most commonly reported sources of GLP-1 influence in our study, campaigns should target platforms such as Instagram, Facebook, YouTube, and TikTok. Search engine optimization (SEO) can also be used to promote credible websites for GLP-1 information. Since LLM-based chatbots such as ChatGPT and Gemini are increasingly being used for online search [55], policy is needed to regulate the information provided by publicly available AI agents. Prompt engineering experiments is one approach that can inform regulatory guidelines by testing how variance in search queries (e.g., including details of medical conditions or demographic factors) impacts responses from AI chatbots. See [56] for example of experimental approach to test accuracy of LLM responses when altering details mentioned in the prompts.

### Limitations & Future Research Directions

There are several limitations that are worth noting for this study. One is generalizability, as there may be a selection bias when recruiting from Amazon Mechanical Turk. While our sample size was determined to adequately observe effects for the conjoint exercise (see OSF link for summary of the conjoint design’s statistical power), our sample is underpowered for generalizing about all GLP-1 users in the US, limiting the certainty of our findings from the exploratory analysis. In particular, our analysis of high-risk purchasers requires further verification, as some effects did not remain significant after adjusting for multiple comparisons. The limited sample size may also produce false negatives, where real differences with smaller effect sizes may be undetected in our analysis. Future work with a larger sample size and screening criteria specific to known patient populations that use GLP-1s is needed to develop and test hypotheses based on our exploratory analysis to make more generalized claims about GLP-1 use.

We were also unable to confirm eligibility (e.g., current GLP-1 use) other than through self-report measures. Researchers should consider partnering with healthcare systems and patient advocacy organizations to validate these findings by confirming eligibility through medical records. However, verifying eligibility in this manner may also exclude participants who are engaged in high-risk sourcing, who should not be overlooked given the multiple reports of falsified GLP-1 products on the Internet and changes in regulatory policy [4,17]. As the focus of our research was to examine *online* purchasing, an online sample may in fact be more appropriate for recruitment than an in-person sample since participation reflects higher digital literacy (i.e., skills required to use the internet and information and communication technologies effectively) and competency with virtual transactions. Older GLP-1 users recruited in clinical settings may not be active online and less likely to make online purchases, making them less relevant for our research objective. Researchers interested in examining effectiveness of marketing strategies toward older GLP-1 users should design and test stimuli adapted to traditional advertising channels (e.g., print magazine, TV commercials).

Outcome metrics based on self-report questions, such as purchasing from high-risk channels, may also be susceptible to measurement error. While the conjoint exercise is designed to mitigate concerns from social desirability bias, the responses to follow-up questions may still be biased from social stigmas associated with purchasing from high-risk sources, unreliable memory recall, and potential misinterpretations of pharmaceutical terms (e.g., legal definition of “online pharmacy”). The existence of platforms and access channels that operate in regulatory “gray areas” further highlights the need for more detailed definitions of sourcing channels to prevent ambiguity. With these limitations in mind, our finding that almost half of GLP-1 users (45.8%, n = 65) have purchased from a high-risk channel may indicate an important public health concern since a social desirability bias suggests participants would *underreport* high-risk purchasing behaviors. Additional work with a larger sample size is needed to verify this finding. If a high proportion of GLP-1 users are in fact purchasing from high-risk channels, then this further highlights the need to increase supply chains and promote educational campaigns on safe online purchasing practices, as discussed in the previous section. When considering that availability of GLP-1 products continue to evolve, including cultural norms surrounding use, policy and enforcement changes regarding marketing and access, future studies may also observe a lower incidence in high-risk purchasing due to increased supply and accessibility.

Another important limitation of our sample is that it skews male and younger, which does not represent typical GLP-1 prescription users. Recent reports indicate that adults aged 50−64 years old are the most likely to report GLP-1 use [53,57], whereas our sample skews younger with an average age of 34.71 (SD = 9.61). The younger age of our sample may reflect a natural fallout from screening criteria based on previously purchasing a health product online. Prior work also reports that women aged 30−49 years old are more than twice as likely to have used GLP-1s than their male peers (15.1% Females vs 7.2% Males) [57]. However, with the exception of this age group, differences from gender use become less pronounced [57] and recent reports show no gender differences in GLP-1 prescriptions for diabetes patients [58,59]. Since older adults have a higher likelihood of needing GLP-1 prescription for medical conditions, this may explain the less pronounced gender differences among older adults, whereas younger GLP-1 users motivated by weight loss may experience more pressure from social norms. Future work should recruit a larger sample of GLP-1 users to conduct more comprehensive analysis on gender and age differences in online purchasing habits. In light of prior work showing that older adults tend to be lower in digital literacy [60,61] and health literacy (i.e., capacity to obtain, process, and understand basic health information and services sufficiently to make appropriate health decisions) [62], these factors should also be examined as potential confounds. In our analysis, we did not detect statistically significant correlations between age and utility scores from the conjoint exercise. However, factors such as digital literacy and health literacy may be more influential on post feature preferences since they directly measure how people navigate online environments and process health-related information, which are necessary skills when evaluating social media posts that advertise GLP-1 drugs. While our sample does not reflect the current demographic composition of GLP-1 users, the growing ubiquity of GLP-1s for weight loss and shifting cultural norms may lead to higher uptake across demographic groups, hence the need for investigating GLP-1 use and perceptions across diverse samples.

As typical with conjoint analysis, tested attributes are dependent on the researcher’s decisions. Therefore, it is possible and likely that we did not test all relevant features of social media posts related to purchasing intent. One important attribute we did not test was price since our focus was examining the influence of online marketing elements. Future work can build on our findings by examining the effects from economic considerations such as pricing and coverage on GLP-1 purchasing decisions. For instance, there may be price thresholds that show when a GLP-1 user is more likely to consider a high-risk seller due to lower costs. Identifying this threshold or specific pricing differentials could set a target price for GLP-1 products from legitimate channels that mitigates the risk of high-risk purchasing. Further, changing dynamics of prescription drug benefit coverage, prescribed indication for use, introduction of new and potentially cheaper formulations, and an individual’s own insurance coverage can significantly impact purchase and sourcing behavior for prescribed drugs and are critical features to understand real-world consumer/patient behavior. Hence, future studies on this topic should attempt to build in pricing and coverage considerations as measures or controls for conjoint and survey-based studies, which in turn, can help in the development of evidence-based policy interventions that reduce high-risk sourcing by identifying primary drivers of purchasing decisions and barriers to legitimate access.

## Conclusions

As demonstrated in the current study, conjoint analysis can account for complexities of consumer decision-making processes within online environments where features of both the product (e.g., branding) and post itself (e.g., caption text) can influence purchase intent. As demand for GLP-1 products will likely continue to rise and the policy landscape changes (e.g., insurance coverage, future affordability, drug shortages, and availability of compounded versions), further effort is needed to promote legal and safe access, especially for those with urgent medical need and who may lose coverage and access.

## Supporting information

S1 Methods Screening Criteria. The following questions were used to screen participants. Participants were eligible if they report currently taking GLP-1 based drugs, have previously taken GLP-1 drugs, or actively looking or considering to try them (Screeners 1 & 2). Participants must also have purchased a consumable health product online (Screener 3). Respondents were removed if they failed at least 2 of the following data quality checks: failing attention checks, straight lined survey questions, provided nonsensical responses to open text questions, and completed the survey at an unusually quick time (10th percentile or less).
(DOCX)

S1 Table Income. (DOCX)

S2 Table Gender. (DOCX)

S3 Table Hispanic. (DOCX)

S4 Table Education. (DOCX)

S5 Table Brand Preference by GLP-1 User Type (Current User, Past User, Considering). (DOCX)

S6 Table Knowledge of GLP-1s by User Type (Current User, Past User, Considering). (DOCX)

S1 Text Interpretation of GLP-1 Knowledge Results in S6 Table. (DOCX)

S1 Fig Websites and Online Platforms used to Ever Purchase GLP-1s. (DOCX)

S2 Fig Influences of GLP-1 Perceptions. (DOCX)

S3 Fig Social Media Platforms that Influenced GLP-1 Perceptions. (DOCX)
