# Supplementary material for: Investigating GLP-1 sourcing preferences on social media and motivations for use: Survey with choice-based conjoint (CBC) exercise
Source: PLoS One. 2026 Nov 4;21(11):e0359937. doi: 10.1371/journal.pone.0359937 (PMC13734441; doi:10.1371/journal.pone.0359937)
Supplement: Supplementary file 1 [file pone.0359937.s001.docx]

**S1 Methods**

**Screening Criteria**
The following questions were used to screen participants. Participants were eligible if they report currently taking GLP-1 based drugs, have previously taken GLP-1 drugs, or actively looking or considering to try them (**Screeners 1 & 2**). Participants must also have purchased a consumable health product online (**Screener 3**). Respondents were removed if they failed at least 2 of the following data quality checks: failing attention checks, straight lined survey questions, provided nonsensical responses to open text questions, and completed the survey at an unusually quick time (10th percentile or less).

**Screener 1**: Have you ever heard of GLP-1 based drugs such as Ozempic or Wegovy?

- Yes (1)
- No (2) **[TERM]**

**Screener 2**: Which of the following best describes your relationship with GLP-1 based drugs such as Ozempic or Wegovy?

- I am currently taking GLP-1 based drugs for weight loss (1)
- I am currently taking GLP-1 based drugs for diabetes treatment (2)
- I used to take GLP-1 drugs but no longer (3)
- I have never taken GLP-1 drugs but actively looking to obtain them (4)
- I have never taken GLP-1 drugs but would consider trying them (5)
- I have never taken GLP-1 drugs and never would (6) **[TERM]**

**Screener 3:** Have you ever purchased a consumable health product ***online*** for yourself or for another person? Health products could be: vitamins, diet pills, weight gain powder, supplements, drugs (prescribed and/or recreational), or medicine of any kind. This includes purchases made via an e-commerce site such as Amazon, an online pharmacy (ex- CVS, RiteAid), social media platforms such as Facebook marketplace or Instagram, messaging apps (e.g. Telegram, WhatsApp, Signal), or other online marketplaces.

- Yes (1)
- No (2) **[TERM]**
- Unsure (3) **[TERM]**
