# Supplementary material for: Investigating GLP-1 sourcing preferences on social media and motivations for use: Survey with choice-based conjoint (CBC) exercise
Source: PLoS One. 2026 Nov 4;21(11):e0359937. doi: 10.1371/journal.pone.0359937 (PMC13734441; doi:10.1371/journal.pone.0359937)
Supplement: Supplementary file 2 [file pone.0359937.s002.docx]

**S1 Table.** Income

| **Income** | **N=, (%)** |
| --- | --- |
| Under $30,000 | N=4, (1.65%) |
| Between $30,000 and $49,999 | N=52, (21.49%) |
| Between $50,000 and $74,999 | N=95, (39.26%) |
| Between $75,000 and $99,999 | N=61, (25.21%) |
| Between $100,000 and $199,999 | N=13, (5.37%) |
| $200,000 or above | N=11, (4.54%) |
| n/a | N=6, (2.48%) |
| *Total* | *242* |
