# Supplementary material for: Investigating GLP-1 sourcing preferences on social media and motivations for use: Survey with choice-based conjoint (CBC) exercise
Source: PLoS One. 2026 Nov 4;21(11):e0359937. doi: 10.1371/journal.pone.0359937 (PMC13734441; doi:10.1371/journal.pone.0359937)
Supplement: Supplementary file 3 [file pone.0359937.s003.docx]

**S2 Table.** Gender

| **Gender** | **N=, (%)** |
| --- | --- |
| Female | N=58, (23.97%) |
| Male | N=176, (72.72%) |
| Transgender Female | N=2, (0.83%) |
| Transgender Male | N=6, (2.48%) |
| *Total* | *242* |
