# Supplementary material for: Investigating GLP-1 sourcing preferences on social media and motivations for use: Survey with choice-based conjoint (CBC) exercise
Source: PLoS One. 2026 Nov 4;21(11):e0359937. doi: 10.1371/journal.pone.0359937 (PMC13734441; doi:10.1371/journal.pone.0359937)
Supplement: Supplementary file 4 [file pone.0359937.s004.docx]

**S3 Table.** Hispanic

| **Response** | **N=, (%)** |
| --- | --- |
| Yes | N=86, (35.54%) |
| No | N=156, (64.46%) |
| *Total* | *242* |
