# Supplementary material for: Investigating GLP-1 sourcing preferences on social media and motivations for use: Survey with choice-based conjoint (CBC) exercise
Source: PLoS One. 2026 Nov 4;21(11):e0359937. doi: 10.1371/journal.pone.0359937 (PMC13734441; doi:10.1371/journal.pone.0359937)
Supplement: Supplementary file 5 [file pone.0359937.s005.docx]

**S4 Table.** Education

| **Education** | **N=, (%)** |
| --- | --- |
| High school graduate (high school diploma or equivalent including GED) | N=4, (1.65%) |
| Some college but no degree | N=1, (0.41%) |
| Bachelor's degree in college (4-year) | N=183, (75.62%) |
| Graduate degree (Masters, PhD, MD, JD, or other doctorate) | N=48, (19.83%) |
| n/a | N=6, (2.48%) |
| *Total* | *242* |
