# Supplementary material for: Investigating GLP-1 sourcing preferences on social media and motivations for use: Survey with choice-based conjoint (CBC) exercise
Source: PLoS One. 2026 Nov 4;21(11):e0359937. doi: 10.1371/journal.pone.0359937 (PMC13734441; doi:10.1371/journal.pone.0359937)
Supplement: Supplementary file 6 [file pone.0359937.s006.docx]

**S5 Table.** Brand Preference by GLP-1 User Type (Current User, Past User, Considering)

|  | ***GLP1 User Type*** | | |
| --- | --- | --- | --- |
|  | ***Current***  (n=142) | ***Past***  (n=28) | ***Consider***  (n=72) |
| **Branded GLP-1 injections/drugs** (e.g., Ozempic, WeGovy, Mounjaro) | 67.6% | 28.6% | 55.6% |
| **Branded GLP-1 semaglutide pills** (e.g., Rybelsus) | 26.1% | 46.4% | *NA* |
| **Generic brand of semaglutide from a compound pharmacy or telehealth service** (e.g., His/Hers, Eden, Ivim) | 5.6% | 25.0% | 36.1% |
| I have **no preference** for branded or generic GLP-1 drugs | 0.7% | 0% | 8.3% |
