# Supplementary material for: Investigating GLP-1 sourcing preferences on social media and motivations for use: Survey with choice-based conjoint (CBC) exercise
Source: PLoS One. 2026 Nov 4;21(11):e0359937. doi: 10.1371/journal.pone.0359937 (PMC13734441; doi:10.1371/journal.pone.0359937)
Supplement: Supplementary file 7 [file pone.0359937.s007.docx]

**S6 Table.** Knowledge of GLP-1s by User Type (Current User, Past User, Considering)

|  | ***Total***  ***Sample***  (n=242) | ***GLP1 User Type*** | | |
| --- | --- | --- | --- | --- |
|  |  | ***Current***  (n=142)  A | ***Past***  (n=28) B | ***Consider***  (n=72)  C |
| I know very little or nothing about GLP-1 drugs | 20.2% | 28.2%  C | 21.4% C | 4.2% |
| I understand the basics about GLP-1 | 49.6% | 52.1% | 39.3% | 48.6% |
| I feel confident enough to make informed choices about GLP-1 drugs | 21.5% | 15.5% | 32.1% | 29.2% |
| I feel totally confident and knowledgeable about GLP-1 drugs | 8.7% | 4.2% | 7.1% | 18.1%  A |

Results are based on two-sided tests. For each significant pair, the key of the category with the smaller column proportion appears in the category with the larger column proportion. Significance level for upper case letters (A, B, C): .05. Tests are adjusted for all pairwise comparisons within a row of each innermost subtable using the Bonferroni correction. *Question Text:* Which of the following best describes your level of understanding when it comes to the benefits of GLP-1 drugs?
