# Supplementary material for: Investigating GLP-1 sourcing preferences on social media and motivations for use: Survey with choice-based conjoint (CBC) exercise
Source: PLoS One. 2026 Nov 4;21(11):e0359937. doi: 10.1371/journal.pone.0359937 (PMC13734441; doi:10.1371/journal.pone.0359937)
Supplement: Supplementary file 8 [file pone.0359937.s008.docx]

**S1 Text. Interpretation of GLP-1 Knowledge Results in S6 Table:**

When asked about knowledge of GLP-1 drugs, almost half of respondents report having a basic understanding (49.6%, n=120) while 20.2% (n=49) state knowing very little or nothing about GLP-1s. Despite using GLP-1 products, current users are 7 times more likely to state knowing very little or nothing about GLP-1 drugs compared to those considering use (28.2% vs 4.2%, p<.05). Conversely, those considering use are over 4 times more likely to report feeling totally confident and knowledgeable about GLP-1 drugs compared to current users (18.1% vs 4.2%, p<.05) These findings suggest that those considering to use GLP-1s may be more likely to formally research the drug before requesting a prescription and implies an educational need for current GLP-1 users.
