# Supplementary material for: Investigating GLP-1 sourcing preferences on social media and motivations for use: Survey with choice-based conjoint (CBC) exercise
Source: PLoS One. 2026 Nov 4;21(11):e0359937. doi: 10.1371/journal.pone.0359937 (PMC13734441; doi:10.1371/journal.pone.0359937)
Supplement: Supplementary file 9 [file pone.0359937.s009.docx]

**S1 Fig.** Websites and Online Platforms used to Ever Purchase GLP-1s.

**Question Text:** Which websites or online platforms have you ever used to purchase GLP-1 drugs? (Select all that apply). Sample is among GLP-1 users who purchased from at least 1 online channel (n=118)
