# Supplementary material for: Investigating GLP-1 sourcing preferences on social media and motivations for use: Survey with choice-based conjoint (CBC) exercise
Source: PLoS One. 2026 Nov 4;21(11):e0359937. doi: 10.1371/journal.pone.0359937 (PMC13734441; doi:10.1371/journal.pone.0359937)
Supplement: Supplementary file 10 [file pone.0359937.s010.docx]

**S2 Fig.** Influences of GLP-1 Perceptions

**Question Text:** Which of the following, if any, have influenced your perception of GLP-1 drugs? Please select all that apply. (n=242)
